# Evaluation of the MIC test strips for antifungal susceptibility testing of *Candidozyma auris* (*Candida auris*) using a representative international collection of isolates

**DOI:** 10.1128/jcm.00399-25

**Published:** 2025-07-03

**Authors:** Maria Siopi, Sevasti Leventaki, Ioannis Pachoulis, Bram Spruijtenburg, Jacques F. Meis, Spyros Pournaras, Georgia Vrioni, Athanasios Tsakris, Joseph Meletiadis

**Affiliations:** 1 Clinical Microbiology Laboratory, Attikon University General Hospital, Medical School, National and Kapodistrian University of Athens 68993, Athens, Greece; 2 Department of Biomedical Sciences, Molecular Microbiology and Immunology Laboratory, University of West Attica, Athens, Greece; 3 Department of Medical Microbiology and Ιmmunology, Canisius Wilhelmina Hospital (CWZ)/Dicoon 6030 , Nijmegen, the Netherlands; 4 Radboudumc-CWZ Center of Expertise for Mycology, Nijmegen, the Netherlands; 5 Institute of Translational Research, Cologne Excellence Cluster on Cellular Stress Responses in Aging-Associated Diseases (CECAD), Excellence Center for Medical Mycology (ECMM), University of Cologne 14309, Cologne, Germany; 6 Department of Microbiology, Medical School, National and Kapodistrian University of Athens, Athens, Greece; University of Utah, Salt Lake City, Utah, USA

**Keywords:** *Candida auris*, MIC test strip, wild type upper limit values, antifungal susceptibility testing, resistance

## Abstract

We compared MIC test strips (MTS) with the reference Clinical and Laboratory Standards Institute (CLSI) broth microdilution method using an international panel of 100 *Candidozyma auris* (*Candida auris*) isolates belonging to different clades. The agreement (±1 twofold dilution) between the methods and the categorical agreement (CA) based on the Centers for Disease Control and Prevention’s (CDC’s) tentative resistance breakpoints and MTS-specific wild-type upper limit values (WT-ULVs) were determined. The MTS-CLSI agreement was poor to weak for posaconazole (3%), itraconazole (20%), voriconazole (31%), and 5-flucytosine (37%), and moderate to strong for isavuconazole (58%), anidulafungin (68%), caspofungin (72%), micafungin (77%), and amphotericin B (85%). Most fluconazole MICs were off-scale, precluding a corresponding estimation. Significant interpretation discrepancies were recorded using the CDC’s breakpoints for amphotericin B (66% CA, 34% major errors; MaEs), but not for fluconazole (98% CA, 1% MaEs, 1% very major errors; VmEs), anidulafungin (97% CA, 3% MaEs, 0% VmEs), micafungin (99% CA, 1% MaEs, 0% VmEs), and caspofungin (95% CA, 5% MaEs, 0% VmEs). Discrepancies were minimized using the amphotericin B method-specific WT-ULV of 4 mg/L (98% CA, 2% MaEs). The MTS-specific WT-ULVs of echinocandins could help to detect 100% of *FKS1* mutants. MTS generated higher MICs than the CLSI for azoles and 5-flucytosine. MTS could accurately detect fluconazole and echinocandin resistance among *C. auris* isolates. Nevertheless, it overestimated amphotericin B resistance as per the CDC’s breakpoint of 2 mg/L. This can be improved by using the MTS-specific WT-ULV of 4 mg/L.

**IMPORTANCE:**

*Candidozyma auris* (*Candida auris*) may exhibit resistance to multiple and sometimes even all currently available classes of antifungals. Hence, antifungal susceptibility testing (AFST) is of key importance to guide the clinician in therapeutic decision-making and to detect novel patterns of resistance. Gradient diffusion strips, referred to both Etest and MIC test strip (MTS), are broadly used in laboratory routine for AFST of yeasts. We therefore compared MTS with the reference Clinical and Laboratory Standards Institute (CLSI) broth microdilution method using an international panel of 100 *C*. *auris* isolates belonging to different clades. Significant interpretation discrepancies were recorded for amphotericin B (66% categorical agreement, 34% major errors), which could be minimized using the amphotericin B method-specific wild-type upper limit value of 4 mg/L. MTS generated higher MICs than the CLSI for azoles and 5-flucytosine. MTS could accurately detect fluconazole and echinocandin resistance.

## INTRODUCTION

*Candidozyma auris* (*Candida auris*) is a rapidly emerging cause of life-threatening invasive infections and hospital outbreaks globally (1). Indeed, the World Health Organization classified it among the fungal pathogens that pose an urgent threat to public health (2), while both the Centers for Disease Control and Prevention (CDC) and the European Centre for Disease Prevention and Control reported its spread in healthcare settings at an alarming rate (3, 4). In contrast to other *Candida* spp., *C. auris* frequently exhibits resistance to multiple and sometimes even all currently available classes of antifungals (1, 5, 6), a feature especially disturbing, given the limited antifungal arsenal. Of note, several clinical cases in which antifungal resistance was acquired *in vivo* have been described, indicating the therapy-induced selection of mutant *C. auris* strains (7–10). Hence, antifungal susceptibility testing (AFST) is of key importance to guide the clinician in therapeutic decision-making and to detect novel patterns of resistance (11).

Gradient diffusion strips (GDS), referred to both Etest and MIC test strip (MTS) hereafter, contain a predefined, dried gradient of an antifungal drug and are broadly used in laboratory routine for AFST of yeasts. However, previous studies have demonstrated variable levels of agreement between the GDS technique and the reference broth microdilution (BMD) methodology depending on the *Candida* spp., antifungal agent, and incubation time (12). Currently, comparative evaluations of the GDS and BMD methods for *C. auris* AFST rely on testing isolates derived from restricted geographical areas and are characterized by clade-dependent results (13–17).

Notably, commercially available products for AFST do not always correlate with the standardized BMD method, leading to incorrect classifications when the breakpoints of the latter are applied. Given that the GDS MIC distributions may be different than the BMD MIC distributions, GDS-specific epidemiological cutoff values (ECVs) have been determined for different *Candida* spp. and antifungal drugs (18). To date, tentative fluconazole, amphotericin B, and echinocandin breakpoints for detecting resistance in *C. auris* with the Clinical and Laboratory Standards Institute (CLSI) protocol have been recommended by the CDC (19), whereas species-specific BMD ECVs have been proposed (20, 21). Moreover, *C. auris*-specific wild-type upper limit values (WT-ULVs) for Sensititre YeastOne and amphotericin B (22), as well as for Vitek 2 and most antifungal agents included in the AST-YS08 cards (23), have recently been estimated. Still, no GDS-specific interpretive criteria are available for *C. auris* versus any antifungal agent.

Based on these grounds, we assessed the MTS performance of *C. auris* AFST compared to the reference CLSI BMD method using an international collection of well-characterized isolates selected to cover various susceptibility phenotypes, aiming to evaluate the accuracy of MTS MICs in the global context and to determine method-specific WT-ULVs.

## RESULTS

### CLSI AFST

**Fig 1:**
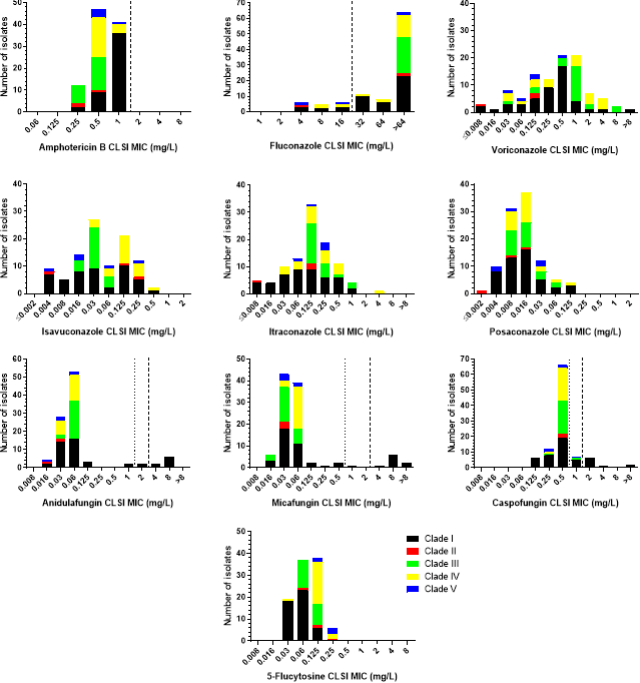
CLSI MIC distributions of *C. auris* isolates by clade. The broken and dotted lines indicate the CDC’s tentative resistance breakpoints and CLSI ECVs for *C. auris* (where available), respectively (19, 21).

The CLSI MIC distributions of *C. auris* isolates by clade are depicted in Fig. 1, while the modal (range) CLSI MICs for each clade separately and all isolates are shown in Table 1. Narrow unimodal MIC distributions were observed for amphotericin B and 5-flucytosine (3–4 twofold dilutions), regardless of clade. All isolates were interpreted as amphotericin B-non-resistant, whereas the modal MIC of clade I strains was slightly higher than the modal MICs of strains from the other clades (1 versus 0.25–0.5 mg/L). Clade I and III isolates had a slightly lower 5-flucytosine modal MIC than the modal MIC of clade IV and V isolates (0.06 versus 0.125–0.25 mg/L).

**TABLE 1:** Clade-specific CLSI and MTS MIC data for *C. auris* isolates obtained in the present study^*e*^

Antifungal agent	Clade (No of isolates)	Modal (range) MIC (mg/L)^*a*^		Median (range) difference CLSI-MTS^*b*^	% agreement		CDC BP (mg/L)	% CA (MaE, VmE) based on CDC BP
		CLSI	MTS		±1	±2	R ≥	
Amphotericin B	All (100)	0.5 (0.25–1)	1 (0.125–>32)	1 (−2 to 6)	85%	96%	2	66% (34%, 0%)
	I (47)	1 (0.25–1)	2 (0.25–>32)	1 (−1 to 6)	83%	91%	2	32% (68%, 0%)
	II (3)	0.25 (0.25–0.5)	0.125 (0.125–0.5)	−1 (−2 to 1)	67%	100%	2	100% (0%, 0%)
	III (23)	0.5 (0.25–0.5)	0.5 (0.5–1)	1 (0 to 2)	83%	100%	2	100% (0%, 0%)
	IV (22)	0.5 (0.5–1)	1 (0.5–2)	1 (−1 to 2)	95%	100%	2	96% (4%, 0%)
	V (5)	0.5 (0.5–1)	0.5 (0.5–2)	0 (−1 to 2)	80%	100%	2	80% (20%, 0%)
5-Flucytosine	All (100)	0.125 (0.03–0.25)	0.25 (0.03–4)	2 (−2 to 6)	37%	69%	NA	ND
	I (47)	0.06 (0.03–0.125)	0.25 (0.06–4)	3 (1 to 6)	9%	43%	NA	ND
	II (3)	ΝD^*c*^ (0.06–0.25)	0.03 (0.03–0.25)	−1 (−2 to 0)	67%	100%	NA	ND
	III (23)	0.06 (0.06–0.125)	0.25 (0.03–2)	1 (−1 to 5)	57%	91%	NA	ND
	IV (22)	0.125 (0.03–0.25)	0.25 (0.25–0.5)	1 (0 to 4)	64%	95%	NA	ND
	V (5)	0.25 (0.125–0.25)	0.5 (0.25–1)	1 (1 to 3)	80%	80%	NA	ND
Anidulafungin	All (100)	0.06 (0.016–8)	0.06 (0.008–>32)	1 (−4 to 6)	68%	79%	4	97% (3%, 0%)
	I (47)	0.06 (0.016–8)	0.06 (0.016–>32)	1 (−4 to 6)	47%	62%	4	94% (6%, 0%)
	II (3)	0.03 (0.016–0.03)	0.06 (0.008–0.06)	1 (−2 to 2)	33%	100%	4	100% (0%, 0%)
	III (23)	0.06 (0.03–0.06)	0.03 (0.016–0.125)	−1 (−2 to 1)	96%	100%	4	100% (0%, 0%)
	IV (22)	0.06 (0.03–0.06)	0.06 (0.03–0.5)	1 (−1 to 4)	86%	91%	4	100% (0%, 0%)
	V (5)	0.03/0.06 (0.016–0.06)	0.03 (0.03–1)	1 (0 to 5)	80%	80%	4	100% (0%, 0%)
Micafungin	All (100)	0.03 (0.016–>8)	0.06 (0.016–>32)	1 (−3 to 7)	77%	91%	4	99% (1%, 0%)
	I (47)	0.03 (0.016–>8)	0.125 (0.016–>32)	1 (−3 to 7)	55%	81%	4	98% (2%, 0%)
	II (3)	0.03 (0.03–0.03)	0.03 (0.016–0.03)	0 (−1 to 0)	100%	100%	4	100% (0%, 0%)
	III (23)	0.03 (0.03–0.06)	0.06 (0.03–0.125)	0 (−1 to 2)	96%	100%	4	100% (0%, 0%)
	IV (22)	0.06 (0.03–0.06)	0.06 (0.06–0.125)	0 (0 to 1)	100%	100%	4	100% (0%, 0%)
	V (5)	0.03 (0.03–0.06)	0.06 (0.016–0.125)	0 (−1 to 2)	80%	100%	4	100% (0%, 0%)
Caspofungin	All (100)	0.5 (0.125–>8)	0.25/0.5 (0.125–>32)	0 (−2 to 6)	72%	85%	2	95% (5%, 0%)
	I (47)	0.5 (0.125–>8)	0.5 (0.125–>32)	1 (−2 to 6)	51%	68%	2	92% (8%, 0%)
	II (3)	0.5 (0.5–0.5)	0.25 (0.125–0.25)	−1 (−2 to −1)	67%	100%	2	100% (0%, 0%)
	III (23)	0.5 (0.25–1)	0.25 (0.25–2)	−1 (−2 to 2)	91%	100%	2	96% (4%, 0%)
	IV (22)	0.5 (0.25–0.5)	0.5 (0.25–1)	0 (−1 to 2)	95%	100%	2	100% (0%, 0%)
	V (5)	0.25/0.5 (0.25–1)	0.25 (0.25–0.25)	−1 (−2 to 0)	80%	100%	2	100% (0%, 0%)
Fluconazole	All (100)	>64 (4–>64)	>256 (1–>256)	ND^*d*^	ND^*d*^	ND^*d*^	32	98% (1%, 1%)
	I (47)	>64 (4–>64)	>256 (1–>256)	ND^*d*^	ND^*d*^	ND^*d*^	32	100% (0%, 0%)
	II (3)	>64 (4–>64)	>256 (1–>256)	ND^*d*^	ND^*d*^	ND^*d*^	32	100% (0%, 0%)
	III (23)	>64 (>64–>64)	>256 (>256–>256)	ND^*d*^	ND^*d*^	ND^*d*^	32	100% (0%, 0%)
	IV (22)	>64 (8–>64)	>256 (8–>256)	ND^*d*^	ND^*d*^	ND^*d*^	32	92% (4%, 4%)
	V (5)	4/>64 (4–>64)	1/>256 (1–>256)	ND^*d*^	ND^*d*^	ND^*d*^	32	100% (0%, 0%)
Voriconazole	All (100)	0.5/1 (≤0.008–>8)	>32 (0.016–>32)	2 (−2 to 9)	31%	52%	NA	ND
	I (47)	0.5 (≤0.008–>8)	0.25 (0.03–>32)	2 (−2 to 9)	40%	70%	NA	ND
	II (3)	0.125 (≤0.008–0.125)	ΝD^*c*^ (0.03–2)	3 (2 to 4)	0%	33%	NA	ND
	III (23)	1 (0.03–8)	>32 (0.5–>32)	6 (−1 to 9)	9%	13%	NA	ND
	IV (22)	1/2/4 (0.03–4)	>32 (0.06– 32)	2 (−1 to 6)	41%	55%	NA	ND
	V (5)	0.125 (0.03–0.125)	0.016 (0.016–>32)	2 (−2 to 9)	20%	60%	NA	ND
Isavuconazole	All (100)	0.03 (0.004–0.5)	0.06 (0.004–2)	1 (−5 to 6)	58%	82%	NA	ND
	I (47)	0.125 (0.004–0.5)	0.016 (0.004–2)	1 (−4 to 6)	57%	79%	NA	ND
	II (3)	ΝD^*c*^ (0.004–0.25)	ΝD^*c*^ (0.004–1)	1 (0 to 2)	67%	100%	NA	ND
	III (23)	0.03 (0.0160.06)	0.06 (0.03–0.5)	1 (0 to 3)	61%	91%	NA	ND
	IV (22)	0.125 (0.03–0.5)	0.5 (0.06–1)	1 (0 to 3)	59%	86%	NA	ND
	V (5)	0.016 (0.004–0.25)	0.008/1 (0.008–1)	1 (−5 to 6)	40%	40%	NA	ND
Posaconazole	All (100)	0.016 (≤0.002–0.125)	0.125 (0.03–1)	4 (1 to 7)	3%	21%	NA	ND
	I (47)	0.016 (0.004–0.125)	0.06 (0.03–1)	3 (1 to 7)	6%	32%	NA	ND
	II (3)	ΝD^*c*^ (≤0.002–0.016)	ΝD^*c*^ (0.03–1)	5 (4 to 7)	0%	0%	NA	ND
	III (23)	0.008/0.016 (0.008–0.06)	0.25 (0.06–0.5)	4 (2 to 5)	0%	9%	NA	ND
	IV (22)	0.016 (0.008–0.125)	0.25 (0.125–1)	4 (2 to 6)	0%	14%	NA	ND
	V (5)	0.004/0.03 (0.004–0.03)	0.06 (0.06–1)	4 (2 to 7)	0%	20%	NA	ND
Itraconazole	All (100)	0.125 (≤0.008–4)	0.25 (0.06–8)	2 (−2 to 8)	20%	52%	NA	ND
	I (47)	0.06/0.125 (≤0.008–1)	0.25 (0.06–4)	2 (−2 to 8)	20%	52%	NA	ND
	II (3)	0.125 (≤0.008–0.125)	ΝD^*c*^ (0.125–4)	4 (4 to 5)	0%	0%	NA	ND
	III (23)	0.125 (0.125–1)	0.5 (0.25–2)	2 (−2 to 3)	26%	74%	NA	ND
	IV (22)	0.125 (0.03–4)	1 (0.5–8)	3 (−2 to 5)	5%	32%	NA	ND
	V (5)	0.25 (0.06–0.25)	ΝD^*c*^ (0.125–2)	2 (−1 to 4)	40%	60%	NA	ND

^*a*^ For bimodal distributions, both modal MICs are presented.

^*b*^ Number of twofold dilutions.

^*c*^ Different MIC value for each isolate precluding the determination of the mode.

^*d*^ Most of the MIC values were off-scale (64/100 isolates with CLSI MIC >64 mg/L and 83/100 isolates with MTS MIC >256 mg/L), precluding the estimation of quantitative agreement.

^*e*^ BP: breakpoint, NA: not available, ND: not determined, R: resistant.

Overall, unimodal MIC distributions were found for echinocandins. Taking into account the different clades, clade I strains demonstrated wider distributions of MIC values compared to the MIC distributions of strains belonging to the other clades (4–5 versus 1–3 twofold dilutions of non-*FKS1* mutants). All WT strains had anidulafungin, micafungin, and caspofungin MICs of 0.016–0.125, 0.016–0.25, and 0.125–1 mg/L, respectively. On the other hand, the *FKS1* mutants (S639F/P/T/Y, M690V, and Δ635F) had anidulafungin, micafungin, and caspofungin MICs of 1 to 8 mg/L, 0.5 to >8 mg/L, and 1 to >8 mg/L, respectively (Table 2). *FKS1* mutants with WT MICs were retested, and their MICs were within ±1 twofold dilution. All *FKS1* mutant isolates displayed additional resistance to fluconazole (MICs >64 mg/L).

**TABLE 2:** Echinocandins’ CLSI and MTS MIC data for the 13 *C*. *auris FKS1* mutant isolates belonging to clade I^*a*^

ID^*b*^	*FKS1* genotype	OD_530nm_ at 24 h	Anidulafungin MIC (mg/L)		Micafungin MIC (mg/L)		Caspofungin MIC (mg/L)	
			CLSI	MTS	CLSI	MTS	CLSI	MTS
AMU129	S639F	0.1815	4	>32	8	>32	2	>32
AMU134	S639F	0.2359	8	>32	8	>32	2	>32
AMU135	S639F	0.1764	8	>32	8	8	2	>32
AMU128	S639P	0.1808	1	>32	8	8	2	>32
AUH 2689	S639P	0.2073	4	>32	4	>32	8	>32
AMU132	S639P	0.2213	8	>32	8	>32	4	>32
AMU111	S639T	0.1991	2	>32	1	0.5	1	>32
AMU133	S639Y	0.2411	2	>32	0.5	>32	1	>32
AMU137	M690V	0.2167	8	>32	8	>32	2	>32
AMU130	Δ635F	0.2358	8	>32	>8	>32	>8	>32
AMU131	Δ635F	0.1853	8	>32	>8	>32	>8	>32
	No of resistant isolates (%) (CDC BP)		8 (73%)	11 (100%)	9 (82%)	10 (91%)	9 (82%)	11 (100%)
	No of non-WT isolates (%) (CLSI ECV)		10 (91%)	11 (100%)	10 (91%)	10 (91%)	11 (100%)	11 (100%)
	No of non-WT isolates (%) (MTS WT-ULV)		ND	11 (100%)	ND	11 (100%)	ND	11 (100%)

^*a*^ Shaded cells indicate resistance based on the CDC’s breakpoints (BP) (anidulafungin/micafungin/caspofungin 4/4/2 mg/L) (19). Underlined numbers indicate wild-type (WT) phenotype based on the CLSI epidemiological cutoff values (ECV) (anidulafungin/micafungin/caspofungin 1/0.5/0.5 mg/L) (21). All isolates were non-WT based on the MTS wild-type upper limit value (WT-ULV) (anidulafungin/micafungin/caspofungin 0.25/0.25/2 mg/L) determined in the present study. ND: not determined.

^*b*^ AMU; Attikon Mycology Unit, AUH: Attikon University Hospital.

In total, the MIC distributions of azoles spanned ≥6 to ≥ 12 twofold dilutions (the exact number of dilution steps could not be determined because of off-scale MICs). In addition, more than one peak was observed for voriconazole and isavuconazole MIC distributions (4 and 2 peaks, respectively), as opposed to the unimodal itraconazole and posaconazole MIC distributions. However, clade-specific patterns were detected. In particular, multimodal MIC distributions were observed for voriconazole and clade IV strains, isavuconazole and clade I and II strains, itraconazole and clade I strains, and posaconazole and clade II, III, and V strains. The number of peaks could not be evaluated for fluconazole because of high off-scale MICs. All clade III isolates were fluconazole resistant (MIC >64 mg/L), while the fluconazole resistance rates of clade I, II, IV, and V strains were 83%, 67%, 77%, and 40%, respectively (overall 83%).

The absolute inter-observer agreement was excellent (96%), while the MIC values for the quality control strains were within the reference ranges.

### MTS AFST

**Fig 2:**
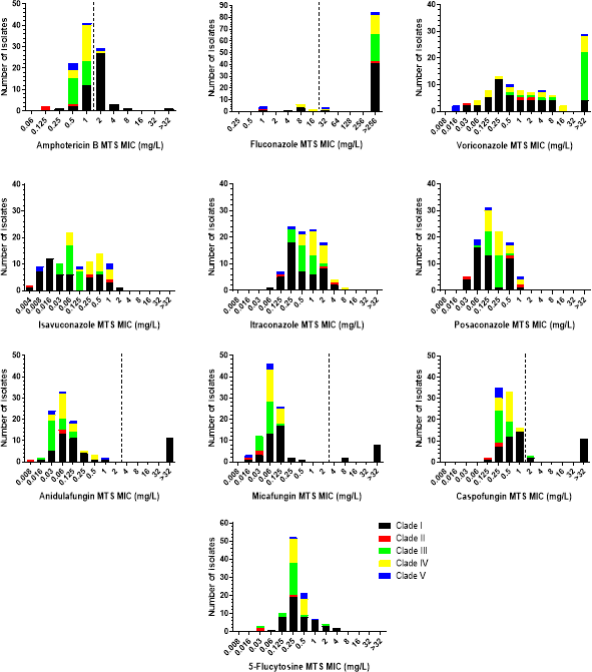
MTS MIC distributions of *C. auris* isolates by clade. The broken lines indicate the CDC’s tentative resistance breakpoints for *C. auris* (where available) (19).

The MTS MIC distributions of *C. auris* isolates by clade are presented in Fig. 2, whereas the modal (range) MTS MICs for each clade separately and all isolates are shown in Table 1. The amphotericin B MIC distribution was unimodal, regardless of clade, although clade-specific patterns were found, with MIC ranges of clade I strains spanning ≥9 twofold dilutions (6 two-fold dilutions if the isolate with an MTS MIC >32 mg/L is excluded), as opposed to the narrow MIC ranges of strains from the other clades (2–3 twofold dilutions). Moreover, clade II isolates had lower MICs (modal MIC 0.25 mg/L) than clade I isolates (modal MIC 2 mg/L), and clade III, IV, and V strains had in-between MICs (modal MIC 0.5–1 mg/L). Hence, amphotericin B resistance rates varied from 0% for clade II and III isolates to 5%, 20%, and 68% for clade IV, V, and I isolates, respectively.

With regards to 5-flucytosine, a unimodal MIC distribution was observed, regardless of clade, with clade I and III strains demonstrating wider distributions of MIC values compared to the MIC distributions of strains from the other clades (7 versus 2–4 twofold dilutions). In addition, clade II isolates had a lower modal MIC than the modal MIC of the other clades (0.03 versus 0.25–0.5 mg/L).

Concerning the echinocandins, the MIC distributions were unimodal, regardless of clade, with clade I strains showing wider distributions of MIC values compared to the MIC distributions of strains from the other clades (4–7 versus 1–4 twofold dilutions, excluding the *FKS1* mutants). Clade I and IV isolates had a slightly higher anidulafungin and caspofungin modal MIC than the modal MIC of the other clades (0.06 versus 0.03 mg/L and 0.5–1 versus 0.25 mg/L, respectively), whereas the micafungin modal MIC of clade II strains was slightly lower than the modal MICs of strains from the other clades (0.03 versus 0.06–0.125 mg/L). All non-*FKS1* mutants had anidulafungin, micafungin, and caspofungin MTS MICs 0.008–1, 0.016–0.25, and 0.125–2 mg/L, respectively. On the contrary, the *FKS1* mutants had anidulafungin, micafungin, and caspofungin MICs of 1 to >32, 0.5 to >32 and >32 mg/L, respectively (Table 2). The *FKS1* mutant with a WT micafungin MIC was retested, and its MIC remained unchanged. All *FKS1* mutant isolates exhibited additional resistance to fluconazole (MICs >256 mg/L), while the majority (9/11) also demonstrated resistance to amphotericin B (MICs 2–4 mg/L).

Regarding the azoles, the MIC ranges of voriconazole, isavuconazole, itraconazole, and posaconazole were wide, spanning 6–≥13 twofold dilutions. Multimodal MIC distributions were found for all azoles except posaconazole, for which a unimodal MIC distribution was found. However, clade-specific differences were observed. Voriconazole MIC distributions of clade I, III, and V isolates were unimodal, with clade IV isolates having low MICs (modal MIC 0.016 mg/L), as opposed to clade I isolates (modal MIC 0.25 mg/L) and clade III isolates (modal MIC >32 mg/L). Similarly, the itraconazole MIC distributions of clade III and clade IV strains were unimodal, with clade IV strains having a one-step-higher modal MIC than the modal MIC of clade III strains (1 versus 0.5 mg/L). Isavuconazole MIC distributions of all but clade III isolates (modal MIC 0.06 mg/L), as well as posaconazole MIC distributions of clade I and clade II isolates, were multimodal. The number of peaks could not be evaluated for fluconazole because of high off-scale MICs. All clade III strains were fluconazole resistant (MIC >256 mg/L), while the resistance rates varied from 60% and 67% for clade V and II strains to 77% and 89% for clade IV and I strains, respectively (overall 83%).

The absolute/±1 log_2_ dilution inter-observer and inter-experimental method’s agreement was 51%/88% and 60%/100%, respectively, with the categorical agreement (CA) between both the blinded readers and the independent replicates being excellent (100%) for amphotericin B, fluconazole, and echinocandins. The MTS MIC values for the quality control isolates were within the expected ranges.

### CLSI versus MTS methods and detection of resistance

#### Amphotericin B

**Fig 3:**
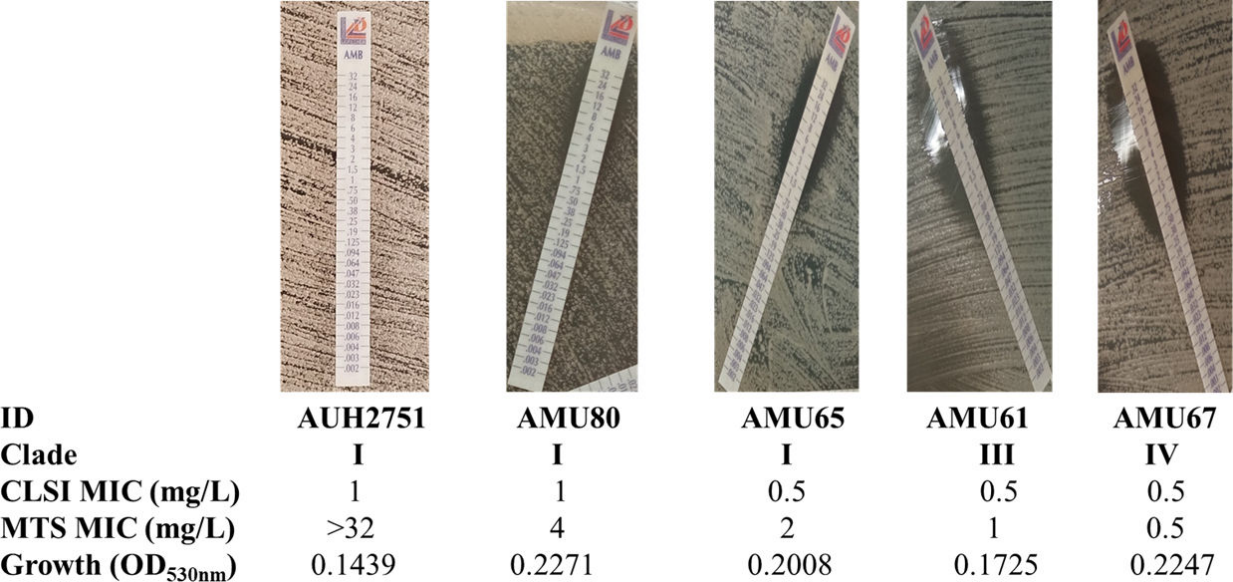
Representative photos for *FKS1* WT *C. auris* isolates with increasing MTS MICs despite similar CLSI MICs. Note that the largest difference between MTS and CLSI was found for isolate AUH2751, which had the lowest OD_530nm_ at 24 h, indicating poor growth in liquid media.

**Fig 4:**
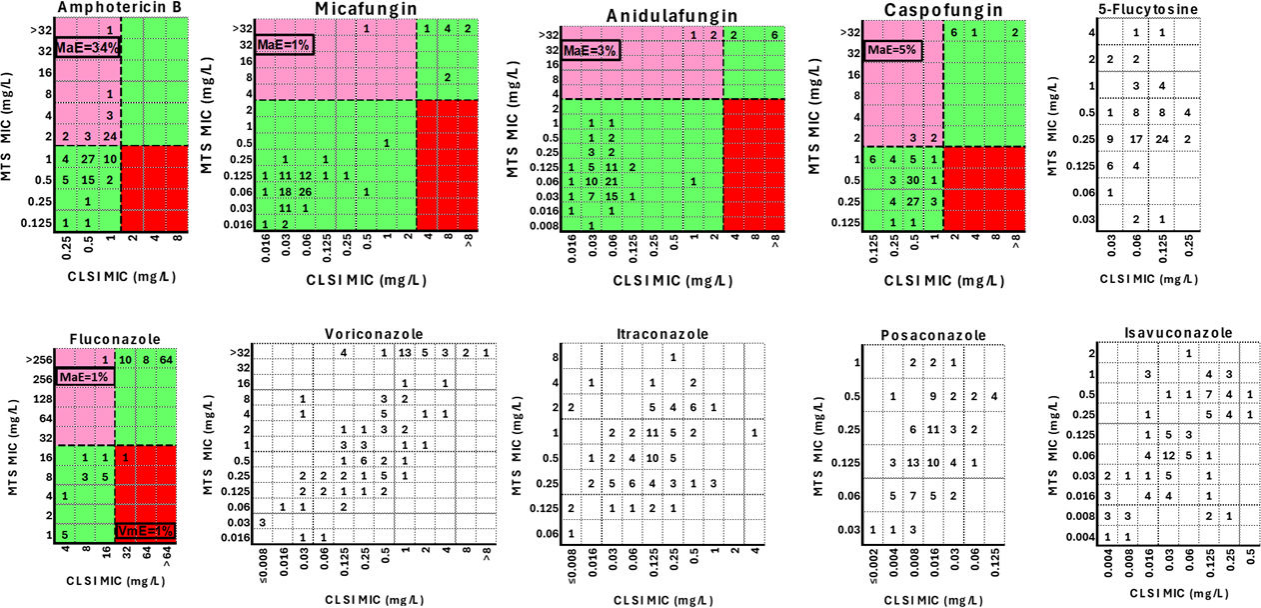
Scatter plots of CLSI MICs versus MTS MICs. Numbers represent the number of *C. auris* isolates (total *n* = 100) at each MIC pair. The black broken lines indicate the CDC’s tentative resistance breakpoints for *C. auris* (where available) (19). The green shaded areas represent categorical agreement, while the pink and red areas indicate major error (MaE) and very major error (VmE), respectively.

The CLSI-MTS agreement within ±1 twofold dilution was strong (85%), with a median (range) difference of 1 (−2 to 6) twofold dilution. The 6 twofold differences between CLSI and MTS MICs were found only for one isolate (AUH 2751), which was reproducible in independent replicates. This isolate reached a lower OD_530nm_ at 24 h compared to other isolates (Fig. 3). Differences for all other isolates ranged from −2 to 2 twofold dilutions. Excellent agreement was found within ±2 twofold dilutions (96%) (Table 1). Although the MIC values generated with these two methods were significantly different (*P* < 0.0001), they demonstrated significant but moderate correlation (Pearson *r* 0.52, *P* < 0.0001), indicating that the MTS MIC distribution is shifted by an average of one twofold dilution higher than the CLSI MIC distribution. Albeit all isolates were interpreted as non-resistant based on the CLSI MICs, 34/100 strains were classified as resistant based on MTS. The corresponding CA was 66% (Cohen’s kappa coefficient (*κ*) =0; no agreement) with 34% major errors (MaEs) (Table 1; Fig. 4).

#### 5-Flucytosine

The CLSI-MTS agreement within ±1 twofold dilution was weak (37%), with a median (range) difference of 2 (−2 to 6) twofold dilutions. Moderate agreement was found within ±2 twofold dilutions (69%) (Table 1). The MIC values obtained via the two methods were significantly different (*P* < 0.0001) and were not correlated (Pearson *r* 0.15, *P* = 0.07). As there are no MIC interpretative criteria for detecting resistance, CA was not determined.

#### Echinocandins

The CLSI-MTS agreement within ±1 twofold dilution was moderate for anidulafungin (68%), micafungin (77%), and caspofungin (72%), with a median (range) difference of 1 (−4 to 6), 1 (−3 to 7), and 0 (−2 to 6) twofold dilution, respectively. Higher agreement was found within ±2 twofold dilutions (79%, 91%, and 85% for anidulafungin, micafungin, and caspofungin, respectively) (Table 1). Despite the fact that the MIC values of all three echinocandins, obtained with the two methods, were significantly different (*P* < 0.006), they showed very strong correlation for anidulafungin and micafungin (Pearson *r* 0.87 and 0.91, respectively, *P* < 0.0001), and significant but moderate correlation for caspofungin (Pearson *r* 0.64, *P* < 0.0001).

If the CLSI BMD was selected as the gold standard, the CAs for anidulafungin, micafungin, and caspofungin were 97% (3% MaEs, 0% VmEs, *κ* = 0.83 [95% CI 0.64–1]; strong agreement), 99% (1% MaEs, 0% VmEs, *κ* = 0.94 [95% CI 0.83–1]; almost perfect agreement), and 95% (5% MaEs, 0% VmEs, *κ* = 0.76 [95% CI 0.55–0.96]; moderate agreement), respectively. The aforementioned interpretation discrepancies for all three echinocandins occurred mainly for isolates belonging to clade I (Table 1; Fig. 4). However, 0–1/11 (0%–9%) isolates with *FKS1* mutations had MTS MICs below the respective CDC resistance breakpoints for anidulafungin, micafungin, and caspofungin. The corresponding levels for CLSI were higher, 18%–27% (Table 2).

#### Azoles

The CLSI-MTS agreement within ±1 twofold dilution was poor to weak for posaconazole (3%), itraconazole (20%), and voriconazole (31%), with a median difference of 4 (1 to 7), 2 (−2 to 8), and 2 (−2 to 9) twofold dilutions, respectively. On the contrary, the agreement was moderate for isavuconazole (58%), with a median difference of 1 (−5 to 6) twofold dilution. Greater agreement was found within ±2 twofold dilutions (posaconazole 21%, itraconazole/voriconazole 52%, and isavuconazole 82%) (Table 1). The MIC values generated with the two methods were significantly different (*P* < 0.0001) and showed significant but moderate correlation (Pearson *r* 0.34–0.68, *P* ≤ 0.0003). As there are no MIC interpretative criteria for detecting resistance, CA was not determined.

For fluconazole, most of the MIC values were off-scale (64/100 isolates with CLSI MIC >64 mg/L and 83/100 isolates with MTS MIC >256 mg/L), precluding the corresponding agreement estimation. All but one of the CLSI fluconazole-non-resistant isolates were correctly classified as such with the MTS method, whereas one CLSI fluconazole-resistant isolate was incorrectly interpreted as fluconazole-non-resistant with the MTS, corresponding to 98% CA (*κ* = 0.93, 95% CI 0.83–1; almost perfect agreement), 1% MaEs, and 1% VmEs. Both of the abovementioned interpretation discrepancies occurred for clade IV strains (Table 1; Fig. 4).

### MTS WT-ULVs

The estimated MTS-specific WT-ULV was 4 mg/L for amphotericin B, 1 mg/L for 5-flucytosine, 0.25 mg/L for anidulafungin, 0.25 mg/L for micafungin, and 1 mg/L for caspofungin (Table 3). WT-ULVs for azoles were not determined as most isolates had high off-scale MICs of fluconazole, and for the other azoles, MIC distributions were multimodal, indicating isolates with different resistance mechanisms. As different modal MICs were found for each clade as described above, the determined WT-ULVs may reflect differences among clades rather than a true WT population.

**TABLE 3:** MTS statistical and visual wild-type upper limit values (WT-ULVs) for the 100 *C*. *auris* isolates used in the present study^*b*^

Antifungal agent	WT-ULV via visual eyeball method (mg/L)	Statistical WT-ULV at indicated endpoint (mg/L)					CLSI ECV (mg/L)
		95%	97.5%	99%	99.5%	99.9%	
Amphotericin B 5-Flucytosine	4 1	4 0.5	4 0.5	4 1	4 1	8 1	2^*a*^ NA
Anidulafungin	0.25	0.125	0.25	0.25	0.25	0.5	1
Micafungin	0.25	0.125	0.25	0.25	0.25	0.25	0.5
Caspofungin	2	1	1	2	2	2	0.5

^*a*^ Based on ECOFFinder 99% ECV determined in a single-center study (20).

^*b*^ ECV: epidemiological cutoff value, ND: not determined, NA: not available.

Based on the MTS-specific amphotericin B WT-ULV of 4 mg/L, CA increased to 98% with only two false-resistant isolates. Based on MTS-specific WT-ULVs of echinocandins, all *FKS1* mutants had anidulafungin, micafungin, and caspofungin MTS MICs higher than the WT-ULVs (Table 2).

## DISCUSSION

The comparative evaluation of MTS and CLSI reference BMD methodologies for AFST against an international panel of *C. auris* isolates indicated that the commercial assay showed drug-related performance patterns. Overall, amphotericin B MTS MICs were slightly higher (modal MIC 1 mg/L) than CLSI MICs (modal MIC 0.5 mg/L); however, notable interpretation discrepancies were observed (34% MaEs, 0% VmEs), which were minimized when a MTS-specific WT-ULV of 4 mg/L was applied (2% MaEs, 0% VmEs). On the contrary, the MTS method could be reliably used for susceptibility testing of fluconazole (1% MaEs, 1% VmEs) and echinocandins (95%–99% CA, 1%–5% MaEs, and 0% VmEs). Some *FKS1* mutants were missed using either the CDC’s breakpoints or CLSI ECVs, as their MICs overlapped with WT isolates or were below the resistance breakpoints. The CLSI-MTS agreement within ±1 twofold dilution was poor to moderate for the rest of azoles (3%–58%) and weak for 5-flucytosine (37%), with MTS resulting in higher MICs.

Studies on antifungal susceptibility patterns of *C. auris*, which have primarily been conducted following the reference BMD methodology, indicate that the MIC distributions can differ significantly among isolates belonging to different clades as found in the present study, although the resistance rate order of fluconazole>amphotericin B>echinocandins seems consistent (1). In fact, such variations may be attributed to differences in the cell morphology and the distinct metabolic properties of each clade-specific *C. auris* phenotype (24–26). Given the variable regional patterns of antifungal resistance, clade-dependent performance of the GDS methods for *C. auris* AFST has been reported, for example, 100% CA for clade II isolates with Etest (17), 86% CA (87% agreement within ±2 twofold dilutions) for clade I isolates with Etest (16), and 86% CA (39% agreement ±2 twofold dilutions) for clade III isolates with MTS (15). A possible explanation for the aforementioned variation could be the clade-specific differences in amphotericin B MIC distributions. In particular, the modal MIC for clade II isolates was 0.5 mg/L (17) as opposed to 1 mg/L for clade I strains, just one twofold dilution step lower from the CDC’s tentative resistance breakpoint of 2 mg/L (16), and the MIC_50_ (MIC range) for clade III isolates was 0.125 (0.06–0.25) mg/L for European Committee on Antimicrobial Susceptibility Testing (EUCAST) and 1 (0.016–2) mg/L for MTS (15).

Considering that treatment with liposomal amphotericin B administered at high doses is recommended as an alternative therapeutic option for *C. auris*-related infections (27), an issue of particular importance is the misleading elevated amphotericin B MTS MICs found for a significant number of strains tested in the present study, leading to 66% CA (34% MaEs). Indeed, Arendrup et al. have recently shown that isolates belonging to three clades exhibited amphotericin B resistance rates of 8%–28% and 45%–63% based on CLSI and Etest MICs, respectively (13), corroborating our findings. Of note, our study revealed that this phenomenon was more prominent among clade I isolates (agreement within ±1/±2 twofold dilutions 83%/91%), resulting in only a 32% CA (68% MaEs). This could be due to the higher MTS MIC values of clade I strains compared to isolates belonging to other clades (modal MIC 2 mg/L versus 0.125–1 mg/L), which is consistent with previous observations using Etest (28, 29). On the other hand, Kathuria et al. reported 86% CA for clade I isolates, most clonal (16), although the evaluation was performed using antibiotic medium three agar plates, a medium that tends to provide slightly lower amphotericin B Etest MICs for *Candida* spp. compared to RPMI (30), whereas lot-to-lot and brand-to-brand variability cannot be excluded, as it is not standardized (31). Clade II isolates were not falsely misclassified with MTS as amphotericin B-resistant (100% CA), as previously described for Etest (17), since they tend to have lower MICs than other clades, while the MTS could be a reliable tool for AFST against clade IV isolates (95% CA), which is in line with previous findings using Etest (14). Isolates with known resistance mechanisms were not included for the comparative evaluation. Yet, the described mechanisms contributing to clinical resistance to amphotericin B in *C. auris* are currently limited (5, 10), whereas there are indications that it may be epigenetic, and thus, the MICs may be reduced after a passage of the isolates in the laboratory with no drug pressure (32).

Unimodal amphotericin B CLSI MIC distributions for *C. auris* have been described (1), supporting our results and revealing a relatively low resistance rate, which is also corroborated by a meta-analysis indicating an overall level of resistance of 12% (33). Nonetheless, a resistance rate of 30% (modal MIC and MIC_90_ values of 0.5 and 2 mg/L, respectively) was found when the susceptibility pattern of 85 Colombian isolates was determined using Etest (34). The corresponding results obtained for strains isolated in the USA (*n* = 200) (35) and Kuwait (*n* = 314) (36) were more pronounced, as the resistance rates according to Etest-generated susceptibility data were 46% (modal MIC and MIC_90_ values of 1 and 2 mg/L, respectively) and 64% (modal MIC and MIC_90_ values of 2 and 4 mg/L, respectively), respectively. Alarmingly, overestimation of amphotericin B resistance in *C. auris* has also been reported with other widely used commercial AFST assays (22, 23), including Etest (37). This discrepancy has been endorsed by both the CLSI (38) and the EUCAST (39) and could be overcome with method-specific ECVs. Notably, amphotericin B resistance would be diminished to 0%, 3%, and 1% for the aforementioned strains, respectively, adopting the MTS-specific amphotericin B WT-ULV of 4 mg/L proposed here.

Furthermore, we incorporated echinocandin-resistant (*FKS1* mutant) strains, as opposed to all previous published studies (14–16). Indeed, echinocandins are currently considered to be the frontline agents for the treatment of infections due to *C. auris* (27), while breakthrough infections, mainly catheter and urinary tract-related, were associated with *FKS1* mutants becoming more frequent (7–9). Therefore, echinocandin AFST data may play a key role in potentially effective therapeutic management, and thus, it is critical to test a collection of truly resistant and non-resistant strains, if the true performance of an AFST assay is sought. Previous evaluations have shown a 61%/84% EUCAST-MTS agreement within ±1/±2 twofold dilutions for anidulafungin and 84%/94% for micafungin (100% CA for both drugs) (15), a 100% CA between CLSI and Etest for micafungin (14) and a CLSI-Etest CA of 99%–100% (0%–1% VmEs) for caspofungin (14, 16), which are in concordance with our results. However, when studies are performed using an abundance of susceptible/WT isolates, as in the latter (14–16), VmEs are uncommon (40), and thus, these results should be interpreted cautiously with respect to whether the method is suitable for the identification of resistant/non-WT isolates. MTS was also able to detect all *FKS1* mutants, with MTS MICs being higher than the CLSI MICs for those isolates.

Moreover, the MTS better discriminated between *FKS1* WT and mutant isolates than the reference BMD technique using the CDC’s breakpoints, as previously also described for *C. albicans* using Etest (41, 42). However, it should be noted that the currently available CDC’s resistance breakpoints are tentative and were not defined based on clinical outcome data (19). Indeed, isolates carrying a *FKS1* mutation have been associated with poor *in vivo* response to echinocandins despite having CLSI MICs below the CDC’s resistance breakpoint (43), suggesting a review of existing echinocandins’ tentative breakpoints for *C. auris* (19). CLSI ECVs or MTS WT-ULVs would help to detect better *FKS1* mutants.

Although the majority (>87%) of *C. auris* strains show resistance to fluconazole (1), non-resistant isolates have been recorded, especially within the clade II and to a lesser extent within the clades I and IV (32). The MTS method allowed correct discrimination between fluconazole-resistant and -non-resistant isolates (98% CA, 1% MaEs, and 1% VmEs), as previously described for GDS (14, 15). Nevertheless, one should keep in mind that persistent or breakthrough *C. auris* candidemias caused by fluconazole-non-resistant isolates (CLSI MICs 2-8 mg/L) have been reported (44), questioning the CDC’s tentative resistance breakpoint of 32 mg/L (19) and discouraging the administration of fluconazole as a therapeutic approach for most clinical cases of *C. auris*-driven infection (45).

To date, the use of fluconazole susceptibility profile is recommended as a surrogate marker for second-generation triazole susceptibility assessments (19). Voriconazole, isavuconazole, itraconazole, and posaconazole CLSI MIC data are comparable with those previously recorded, showing high MIC values and wide distributions (1, 26). In agreement with previous reports using GDS (14–16), a similar picture was observed for our MTS MIC distributions. Overall, the MTS MICs were higher than the CLSI MICs. Head-to-head comparisons of the MICs obtained by the GDS and BMD methods are limited. Kathuria et al. reported a 79% CLSI-Etest agreement within ±2 twofold dilutions for voriconazole (modal Etest MIC 1 mg/L) likely of clonal clade I (Indian) isolates (16), which is in line with our findings. However, Ruiz-Gaitán et al. found a 0%/0% EUCAST-MTS agreement within ±1/±2 twofold dilutions for voriconazole (MTS MIC_50_ >32 mg/L), in contrast to 37%/74% for posaconazole (MTS MIC_50_ 0.25 mg/L) and 69%/88% for isavuconazole (MTS MIC_50_ 0.125 mg/L), possibly of clonal clade III (Spanish) isolates (15). In general, wide MIC ranges typically indicate heterogeneous populations, and thus, most of the *C. auris* isolates should be considered azole-resistant since fluconazole CLSI MICs correlated with CLSI MICs for all other azoles (Pearson *r* 0.29-0.62, *P* ≤ 0.003). This argues for the ineffectiveness of these agents, albeit clinical proof for a relevant MIC-outcome relation is currently lacking (45). If there is a WT population, this will most likely be at the lower end of the MIC distribution. As the MIC distributions of each clade are different, analyzing all clades together will result in wide MIC distributions and high WT-ULVs. However, those values are still higher than the MTS MICs of the first described *C. auris* in 2009 in Japan (0.03 mg/L for posaconazole, 0.004 mg/L for isavuconazole, 0.03 mg/L for voriconazole, and 0.125 mg/L for itraconazole).

Regarding 5-flucytosine, CLSI susceptibility data are consistent with those previously described (modal MIC/MIC_50_ values of 0.125/0.06 mg/L versus 0.06–0.125/0.06–0.125 mg/L, respectively) (26, 46). GDS-generated MIC data sets are scarce. Namely, 5-flucytosine susceptibility testing of 54 *C*. *auris* isolates using Etest demonstrated a narrow MIC range (0.03–0.125 mg/L, modal MIC 0.06 mg/L), with the exception of six strains demonstrating MIC values of 32 mg/L (35), as opposed to our findings (modal [range] MIC 0.25 [0.03–4] mg/L). Of note, the aforementioned strains (clinical, surveillance, and environmental) were obtained from different hospitals during the New York outbreak but were not genotyped to eliminate the presence of clonality, in contrast to our study design. To date, comparative evaluations of the GDS and BMD methods are lacking. Based on our findings, the CLSI-MTS agreement was weak (37% within ±1 log_2_ dilutions), as the MTS method generated higher MICs. A WT-ULV of 1 mg/L was determined in the present study for MTS and 5-flucytosine.

Results of GDS may be affected by a series of methodological issues, like the inoculation method and trailing effects. Although both manufacturers recommend double-dipping for plate inoculation (47), Arendrup et al. have recently shown that the Etest-generated amphotericin B MICs increased when double inoculation was followed compared with single inoculation (modal MIC of 2 versus 1 mg/L, respectively, and resistance rates of 45%–58% versus 25%–30%, respectively, after 24 h of plates’ incubation) (13). Whether this is also the case for the MTS remains unknown. Interestingly, provided that single swab inoculation is adopted, the authors concluded that considering isolates with GDS amphotericin B MIC ≤2 mg/L as WT is a valid option. Nevertheless, following double swabbing inoculation for the Etest method (corresponding data for the MTS were not available), an Etest amphotericin B MIC ≤4 mg/L could enable the discrimination between WT and mutated isolates (13), which is in line with the MTS-specific WT-ULV determined here. Further studies assessing the impact of single versus double inoculation of the agar on the GDS results for *C. auris* and various antifungals are required.

In conclusion, the MTS method enabled the correct categorization of fluconazole- and echinocandin-resistant isolates, detecting all *FKS1* mutants. On the other hand, resistance to amphotericin B was overestimated, whereas the problem was alleviated using the MTS-specific WT-ULV of 4 mg/L. Notably, a key limitation of a study conducted entirely within a single laboratory, such as the present one, is the unknown reproducibility of its findings across other laboratories. Therefore, further multicenter assessments are warranted to corroborate our findings and to determine method-dependent ECVs to streamline the identification of non-WT isolates using GDS-generated MIC data. However, as ECVs are not clinical breakpoints and a WT isolate may or may not respond to therapy, clinical outcome data are needed to set clinical breakpoints.

## MATERIALS AND METHODS

### Isolates

A total of 100 *C*. *auris* isolates were tested. In particular, 17 bloodstream isolates were collected from individual patients hospitalized in 8 Greek tertiary care hospitals located within the Attica region from November 2020 to August 2022. They were identified to the species level using matrix-assisted laser desorption ionization-time of flight mass spectrometry (Bruker Daltonics, Bremen, Germany) and clustered in clade I (South Asian) (48). Moreover, 83 genetically distinct isolates belonging to five *C. auris* clades and being isolated from various geographical regions, namely *n* = 30 clade I (South Asian; Brazil, Kuwait, Iran, India, Oman, Pakistan), *n* = 3 clade II (East Asian; South Korea, Japan), *n* = 23 clade III (African; South Africa, Spain), *n* = 22 clade IV (South American; Venezuela, Colombia), and *n* = 5 clade V (Iranian; Iran), were included (48). The strain collection comprised 11 clade I *FKS1* hotspot mutant isolates harboring non-synonymous or deletion mutations (S639F/P/T/Y, M690V, and ΔF635) that are associated with resistance/elevated MICs to echinocandins, detected as previously described (49).

All isolates were stored at −70°C in normal sterile saline with 10% glycerol (AppliChem, Darmstadt, Germany) until use. Prior to testing, they were revived by subculturing them twice onto in-house prepared antimicrobial-free Sabouraud dextrose agar (Oxoid, Athens, Greece) plates at 35°C ± 2°C for 24 h.

### AFST

The BMD AFST was performed according to the CLSI M27A4 protocol guidelines using laboratory-grade pure powders of amphotericin B (Sigma-Aldrich, Athens, Greece), fluconazole (Sigma-Aldrich, Athens, Greece), voriconazole (Pfizer Ltd., Kent, UK), posaconazole (Sigma-Aldrich, Athens, Greece), itraconazole (Sigma-Aldrich, Athens, Greece), isavuconazole (Sigma-Aldrich, Athens, Greece), anidulafungin (Pfizer, CT, USA), micafungin (Astellas Pharma, Tokyo, Japan), caspofungin (Merck & Co., NJ, USA), and 5-flucytosine (Sigma-Aldrich, Athens, Greece). Briefly, twofold serial drug concentrations ranging from 8 to 0.06 mg/L for amphotericin B, 64 to 1 mg/L for fluconazole, 2 to 0.002 mg/L for isavuconazole and posaconazole, and 8 to 0.008 mg/L for the remaining antifungals were used. The microtiter plates were incubated at 35 ± 2°C for 24 h and the MICs, defined as the lowest drug concentration at which complete (for amphotericin B) or ≥50% (for the rest of antifungals) visual fungal growth inhibition compared to the drug-free control well was observed, were determined with the aid of a magnifying mirror (50).

The MTS AFST was performed strictly following the manufacturer’s (Liofilchem, Roseto degli Abruzzi, Italy) instructions. Briefly, each RPMI agar plate (Liofilchem) was inoculated by double-dipping a sterile swab in the standardized yeast suspension (double inoculation). After applying the MTS to the agar surface, the plate was incubated at 35°C ± 2°C for 24 h and the MIC was determined by visual observation as the lowest drug concentration at which the border of the elliptical inhibition intercepted the strip scale using a 100%, 90%, and 80% fungal growth inhibition endpoint for amphotericin B, 5-flucytosine, and azoles/echinocandins, respectively (47). To assess the potential variation in MTS MIC determination, a proportion of isolates (10/100) have been re-tested on different days so as to determine the method’s inter-day reproducibility.

A single lot of RPMI medium, MTS, and RPMI agar plates was used throughout the study. Each isolate was tested by both AFST methods at the same laboratory using a single fungal suspension, which was prepared in sterile saline solution and adjusted to the required concentration. Inoculum density and purity checks were performed on all isolates by spread plate counts on in-house prepared antimicrobial-free Sabouraud dextrose agar plates. The CLSI and MTS MICs were determined by two blinded observers; discordance was arbitrated by a third reader. Testing was repeated by both the BMD and the MTS method for the isolates displaying discordant results, and the repeat result was kept as the final. The recommended *C. krusei* ATCC 6258 and *C. parapsilosis* ATCC 22019 were used as quality control strains for both methods.

### Comparison between the CLSI and the MTS MICs

A head-to-head comparison of the generated MIC data sets, using the CLSI BMD as the reference methodology, was performed. The MTS MICs that fell between the traditional twofold dilution series were rounded up to the next standard upper twofold value. In any case, high off-scale MIC results were converted to the next highest twofold concentration, whereas low off-scale MIC values were left unchanged. For the quantitative analysis, the results of the two AFST methods were analyzed with a paired Student’s t-test after log_2_ transformation of the MIC values. A two-tailed *P* value of < 0.05 was considered to reveal a statistically significant difference. In addition, the levels of CLSI-MTS agreement within ±1 and ±2 twofold dilutions were calculated. For the qualitative analysis, the CA was estimated following the CDC’s tentative resistance breakpoints for *C. auris* (where available), namely amphotericin B ≥2 mg/L, fluconazole ≥32 mg/L, anidulafungin/micafungin ≥4 mg/L, and caspofungin ≥2 mg/L (19), and its strength was assessed by calculating the *κ*. Discrepancies were considered as MaE when the CLSI classified an isolate as non-resistant and the MTS as resistant (false resistance), and VmE when the CLSI categorized a strain as resistant and the MTS as non-resistant (false non-resistance). Regarding the echinocandins, the ability of each method to detect *FKS1* mutants was assessed. Data were analyzed using the statistics software package GraphPad Prism, version 9.0, for Windows (GraphPad Software, San Diego, CA, USA).

### MTS WT-ULV determination

MTS MIC distributions were analyzed with the ECOFFinder program (available at https://www.eucast.org/mic_and_zone_distributions_and_ecoffs) and visually (eyeball method) (51), and the WT-ULV was defined as the upper MIC value where the WT distribution ends. Different endpoints were used (95%, 97.5%, 99%, 99.5%, and 99.9%) for the statistical WT-ULV determination.
